# The Book World of Medicine and Science

**Published:** 1902-11-01

**Authors:** 


### The Book World of Medicine and Science.

The Practitioner's Guide. By J. Walter Carr, M.D.(Lond.), F.R.C.P.; T. Pickering Pick, F.R.C.S.; Alban H. G. Doran, F.R.C.S.; and Andrew Duncan, M.D., B.S.(Lond.), F.R.C.S., M.R.C.P. (London: Longmans, Green, and Co. 1902. Price 21*s.* net.)

The object of this book is that it may serve as a work of ready reference for the guidance of the practitioner, and this object it will serve admirably well. Obviously it is impossible to condense all knowledge into eleven hundred pages. Hence a good deal has had to be left out. But we are not sure that the authors have not exercised as much judgment, and, we may add, been as happy, in their omissions as in their selection of matter for insertion; the result at any rate has been the production of a volume which is handy in size and absolutely full of useful, practical, and up-to-date information. The arrangement of the subjects is alphabetical, but an index is provided and also plenty of cross references, so that it is more easy than is the case in some dictionaries to follow up the ramifications of any particular subject. Little is said about pathology or etiology, the stress of the authors' endeavours having been devoted to a discussion of symptoms, differential diagnosis, and treatment. In view of the considerable and important part of the work of a general practitioner which is occupied with diseases of women, a relatively large space has been devoted to this branch of the medical art. Again, in the hope of making the book a handy work of reference on all diseases for the use of medical men practising abroad, a considerable amount of attention has been given to tropical diseases. On the other hand midwifery has been left out, as also has any special description of those major operations which can be deliberately undertaken, those operations only which the general practitioner might be called upon to perform in an emergency or at a moment's notice having been described in detail. As to the manner in which the authors have performed their task we have nothing but praise to give. We have looked up all kinds of diseases—medical, surgical, gynæcological, diseases of the eye, ear, etc.—and we have in most cases found short, crisp descriptions, with clear directions as to treatment. Perhaps here and there the authors have gone even beyond their promises, as where they describe the operation for removing the kidney. On the other hand there is no account of either iridectomy or enucleation of the eyeball, notwithstanding that the latter operation is again and again referred to under the head of treatment. But, as we have said, we cannot expect everything. It is enough that what there is is good, and that there is a great deal of it. It is certainly a most useful book of reference—up to date, convenient, and thoroughly trustworthy.

Proceedings of the Society for Psychical Research. (London: R. Brimley Johnson. June 1902. Price 4s. 6d. net.)

This number is almost entirely given over to an account of various interviews with a certain Mrs. Thompson of Hampstead whose powers as a "medium" have excited much interest. We do not feel inclined at the present moment to enter into the many abstruse questions to the solving of which the Society for Psychical Research devotes itself, nor can we bring ourselves to accept even the foundations on which its members base some of their hypotheses; and we will at once warn intending students that unless they enter upon the subject with serious, and we may almost say detective, intent they will find the pages of this volume desperately dull reading. At the same time we would recommend a careful study of its pages to those mocking spirits who, notwithstanding their ignorance of its objects, are so ready to pour out their sarcasm upon this society, for they will therein find a fair specimen of the sort of problems, both of fact and of interpretation, which its members seek to investigate. Probably the whole thing is a mare's nest. But to say that is no more convincing to others than—well, than much of the evidence of Mrs. Thompson is to us.

Notes on Medicine for Medical and Dental Students. By W. D. Woodburn, L.D.S. (London: Baillière Tindall and Cox. 1902. Price 3s. 6d. net.)

It is difficult to imagine the state of mind of one who, though so ill-equipped for his task as the writer of this little book evidently is, rushes into print for the edification of his fellows; but at least we may say that, if the smattering of medicine which dental students are now compelled to acquire is to be taken as justifying them in assuming the rôle of teachers on the subject, considerable mischief will be done. The author of this book says in his preface that he claims no originality for these notes, in saying which perhaps he is a little over modest. On the very first page we have evidence of his lack even of ordinary literary power of expression. He defines acceleration of respiration as "A compensatory phenomenon to make up for part of a lung not working; *i.e.* pneumonia." Why *i.e.*? Evidently he wanted to say, "for example in," but what he says is nothing of the sort. On the next page we come upon such expressions as "bronchialtasis," "opncea," "bronchii." Speaking of "V. R.", by which we presume he means "vocal resonance," he describes bronchophony and pectoriloquy, and then goes on: "V. R., with bleeding character ægophony." No doubt economy in type is a thing to aim at, but we doubt whether examiners at the College will approve of the term which heads the paragraph on "acute or loba pneumonia," nor do we think that it will improve the chances of a candidate who wishes to describe the symptoms of "loba pneumonia" to say "herpeolabialis frequent towards the acrui." So much for the first half dozen pages of this wretched book. Let us run on to the nervous system. Here we find that the author has more originality than he suspects. He speaks of "analgeria" and "hyperalgeria" as if these were quite ordinary conditions. Then we are told that in "acute anterior piolo-myelitis" the "febricular lasts for a few days," that "Albetosis resembles chorea," etc., that "Memerer's disease or aural vertigo is a disease of the labyrinth," that hysteria is "most common during the period of sexual maternity," and so on. To appreciate to the full the fine self-confidence of this self-elected teacher we must see him holding the balance between such authorities as Balfour, Duncan, and M'Call Anderson. Describing the various modes of treatment of aneurysm he speaks of "Balfour's by large doses of K.I. (ʒi daily); Duncan's and M'Call Anderson's, by galvanic puncture, etc. K.I. is best." Which may be true enough; but fancy Mr. Woodburn telling us so!

